# The performance of different propensity-score methods for estimating differences in proportions (risk differences or absolute risk reductions) in observational studies

**DOI:** 10.1002/sim.3854

**Published:** 2010-01-27

**Authors:** Peter C Austin

**Affiliations:** a Institute for Clinical Evaluative SciencesToronto, ON, Canada; b Department of Health Management, Policy and Evaluation, University of TorontoON, Canada

**Keywords:** propensity score, observational study, binary data, risk difference, number needed to treat, matching, IPTW, inverse probability of treatment weighting, propensity-score matching

## Abstract

Propensity score methods are increasingly being used to estimate the effects of treatments on health outcomes using observational data. There are four methods for using the propensity score to estimate treatment effects: covariate adjustment using the propensity score, stratification on the propensity score, propensity-score matching, and inverse probability of treatment weighting (IPTW) using the propensity score. When outcomes are binary, the effect of treatment on the outcome can be described using odds ratios, relative risks, risk differences, or the number needed to treat. Several clinical commentators suggested that risk differences and numbers needed to treat are more meaningful for clinical decision making than are odds ratios or relative risks. However, there is a paucity of information about the relative performance of the different propensity-score methods for estimating risk differences. We conducted a series of Monte Carlo simulations to examine this issue. We examined bias, variance estimation, coverage of confidence intervals, mean-squared error (MSE), and type I error rates. A doubly robust version of IPTW had superior performance compared with the other propensity-score methods. It resulted in unbiased estimation of risk differences, treatment effects with the lowest standard errors, confidence intervals with the correct coverage rates, and correct type I error rates. Stratification, matching on the propensity score, and covariate adjustment using the propensity score resulted in minor to modest bias in estimating risk differences. Estimators based on IPTW had lower MSE compared with other propensity-score methods. Differences between IPTW and propensity-score matching may reflect that these two methods estimate the average treatment effect and the average treatment effect for the treated, respectively. Copyright © 2010 John Wiley & Sons, Ltd.

## 1. Introduction

In randomized controlled trials, the effect of treatment on dichotomous outcomes can be reported using a variety of measures of treatment effect: the odds ratio, the relative risk (and the associated relative risk reduction), the absolute risk reduction, and the number needed to treat (NNT) (the reciprocal of the absolute risk reduction). Schechtman [1] argues that both relative and absolute measures should be reported. Cook and Sackett [2] argue that for clinical decision making the NNT is more meaningful than the relative risk, relative risk reduction, or the odds ratio. Jaeschke *et al.* [3] suggest that the odds ratio and the relative risk provide limited information. Finally, Sinclair and Bracken [4] argue that clinically important questions are best addressed using relative risks, relative risk reductions, risk differences, and the NNT. In the face of these proposals, some medical journals require that the NNT be reported for any randomized controlled trial with a dichotomous outcome [5]. Common to all these recommendations is the agreement that limited information is provided by relative measures of treatment effect such as the odds ratio or the relative risk. Furthermore, a common theme is that risk differences and numbers needed to treat can be of greater importance for clinical decision making than are relative risks and odds ratios.

Researchers are increasingly using observational studies to estimate the effect of treatment on outcomes. In non-randomized studies, unlike in randomized trials, treated subjects often differ systematically from untreated subjects. Therefore, outcomes cannot be compared directly between treated and untreated subjects. Statistical methods must be used to adjust for systematic differences between treated and untreated subjects when estimating the effect of treatment on outcomes. Propensity-score methods are increasingly being used to eliminate the impact of treatment-selection bias when estimating the effect of treatments or exposures on outcomes when using observational data. When outcomes are dichotomous, propensity-score methods allow for estimation of risk differences, relative risks, and odds ratios. The performance of different propensity-score methods for estimating relative risks and odds ratios has been extensively studied [6–8]. However, there is a paucity of information about the performance of different propensity-score methods for estimating risk differences. Given the increasing popularity of propensity-score methods and the clinical importance of both the absolute risk reduction and the NNT, it is important to examine the performance of different propensity-score methods for estimating these quantities. Understanding the performance of different propensity-score methods for estimating absolute risk reductions and NNT will allow for the estimation of more clinically meaningful measures of treatment effect using observational data.

The objective of the paper is to examine the performance of different propensity-score methods for estimating risk differences (or absolute risk reductions). The paper is structured as follows. In Section 2, we briefly review different propensity-score methods for estimating risk differences. In Section 3, we describe an extensive series of Monte Carlo simulations to examine the performance of different propensity-score methods for estimating risk differences. In Section 4, we present an empirical case study in which we compare the estimates of the effect of beta-blocker therapy on survival using a large sample of patients hospitalized with heart failure. Finally, in Section 5 we summarize our findings.

## 2. Review of different propensity-score methods

The propensity score is the probability of treatment selection conditional on observed baseline covariates [9, 10]. Rosenbaum and Rubin [9] demonstrated that conditional on the propensity score, the distribution of observed baseline covariates is independent of treatment selection. In this section we briefly review four methods of using the estimated propensity score to estimate absolute risk reductions (i.e. differences in proportions or risk differences). The first three, stratification on the propensity score, matching on the propensity score, covariate adjustment using the propensity score, were proposed by Rosenbaum and Rubin in their initial article on the propensity score [9]. The fourth, inverse probability of treatment weighting (IPTW), using the propensity score was subsequently developed by Rosenbaum [11].

### 2.1. Stratification on the propensity score

Stratification (or subclassification) on the propensity score involves comparing outcomes between treated and untreated subjects within strata defined by the propensity score. The most common approach is to use five, approximately equally sized strata defined by the quintiles of the propensity score. The effect of treatment on outcomes is estimated within each stratum. Stratum-specific treatment effects are then pooled to obtain an overall treatment effect. Rosenbaum and Rubin demonstrated that stratifying on the quintiles of the estimated propensity score eliminates approximately 90 per cent of the bias due to the measured confounders [9].

Assume that *p_T,i_* and *p_C,i_* denote the proportion of treated and untreated subjects, respectively, in the *i*th stratum that experience the event. Then the stratum-specific risk difference can be estimated as Δ_*i*_=*P_T,i_* – *p_C,i_*. The pooled estimate of the risk difference is then equal to 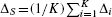, where *K* denotes the number of strata (*K* = 5 when stratification on the quintiles of the propensity score is employed). The variance of each estimated stratum-specific risk difference can be estimated using standard methods for estimating the variance of differences in proportions. The stratum-specific variances can then be pooled to obtain an overall estimate of the variance of the pooled risk difference. Let *n_C,i_* and *n_T,i_* denote the number of untreated and treated subjects in the *i*th strata. Then the variance of Δ_*i*_, can be estimated by


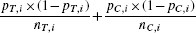


The variance of Δ_*S*_ can be estimated by pooling the stratum-specific variances: 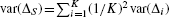.

### 2.2. Matching on the propensity score

Propensity-score matching entails forming matched sets of treated and untreated subjects with similar values of the propensity score. Although there are different approaches to matching, the most common approach in the medical literature is nearest neighbor pair-matching without replacement within specified calipers of the propensity score [12–14]. Using this approach, pairs of treated and untreated subjects are formed such that the propensity score of the matched subjects lies within a specified distance of one another (the caliper width). While a wide variety of calipers have been used in the medical literature [12–14], there has been little research into the relative performance of different propensity-score matching methods [15].

Once a propensity-score matched sample has been formed, the absolute risk reduction can be estimated as the difference between the proportion of untreated subjects experiencing the outcome and the proportion of treated subjects experiencing the outcome in the matched sample. In propensity-score matched analyses, one should account for the matched nature of the sample when estimating the significance of the treatment effect [16]. Thus, the statistical significance of the risk difference can be tested using McNemar's test for correlated binomial proportions [17]. Similarly, confidence intervals for the difference in proportions can be constructed using methods that account for the matched nature of the sample [17]. Assume that in the matched sample, there are *a* pairs in which both the treated and the untreated subjects experience the event; *b* pairs in which the treated subject experiences the event, whereas the untreated subject does not; *c* pairs in which the untreated subject experiences the event, whereas the treated subject does not; and *d* pairs in which both the treated and the untreated subjects do not experience the event. The difference in the probability of the event between the treated and the untreated subjects is estimated by (*b – c*)/*n*, where *n* is the number of matched pairs. The variance of the difference in proportions is estimated by ((*b + c) — (c — b)^2^/n)/n^2^* [17].

### 2.3. Inverse probability of treatment weighting

Let *Z* denote treatment assignment (*Z* = 1 denoting treatment; *Z* = 0 denoting absence of treatment). Furthermore, let *e* denote the propensity score. The inverse probability of treatment is defined as *Z/e*+(1 − *Z*)/(1 − *e*). For each subject, it is equal to the inverse of the probability of receiving the treatment that the subject received. Rosenbaum developed model-based direct adjustment as an extension to conventional direct standardization or adjustment [11]. Model-based direct adjustment weights subjects by the inverse of the probability of treatment. Weighting by the inverse probability of treatment results in a synthetic population in which treatment assignment is independent of measured baseline covariates.

Lunceford and Davidian [18] provide a review of methods for estimating treatment effects that use weighting by the inverse of the probability of treatment. They describe several estimators that use IPTW, of which we describe two in this section. Let *Y_i_* denote the outcome for the *i*th subject, *Z_i_* denote the treatment status of the *i*th subject, whereas *ê_i_* denote the estimated propensity score for this subject. Then the first IPTW estimator of the risk difference, originally proposed by Rosenbaum [11], is


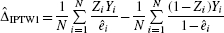
(1)

where *N* denotes the number of subjects in the sample. We refer to the estimator as IPTW1. A second estimator described by Lunceford and Davidian [18] is known as a doubly robust estimator. It requires specifying both the propensity-score model as well as a regression model relating the expected outcome to treatment and baseline covariates. Let *m_z_(**X**, α_*z*_) = *E*(*Y|Z* = *z*, **X**).* Then


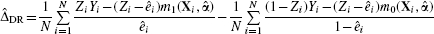
(2)


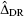
 has a ‘double-robustness’ property in that the estimator remains consistent if either the propensity-score model is correctly specified or if both the outcomes regression models are correctly specified [18]. We refer to this estimator as the IPTW-DR estimator. Lunceford and Davidian provide estimates of the variance for both 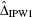 and 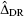.

### 2.4. Covariate adjustment using the propensity score

In covariate adjustment using the propensity score, the effect of treatment on the outcome is estimated by the regression of the outcome on an indicator variable denoting treatment assignment and the propensity score. This is the most commonly used propensity-score method in the medical literature [19, 20]. Logistic regression would be the natural regression model to implement when the outcome is binary. However, with logistic regression the odds ratio, rather than the risk difference, is the measure of effect. One could attempt to use a generalized linear model with a Binomial distribution and the identify link function. Although this would allow for estimation of risk differences, the identify link function does not constrain the predicted probability to lie between 0 and 1. This can lead to computational problems and lack of model convergence in practice. As an alternative, one could use a linear regression model estimated using ordinary least squares (OLS):


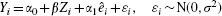
(3)

where *Z_i_* and *ê_i_* denote the treatment assignment and estimated propensity score, respectively, for the *i*th subject. The estimated regression coefficient for the treatment indicator, 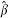, would be used to estimate the reduction in the probability of the outcome due to treatment. The standard error of the estimated difference in proportion could be the model-based estimated standard error of the regression coefficient 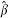. A limitation of this approach is that it ignores the fact that the outcomes are dichotomous. Thus, the predicted probability of the event may not be constrained to lie between 0 and 1. Furthermore, the assumptions for the distribution of the error terms may no longer be satisfied, since the variance of a predicted probability is not uniform but is a function of the proportion itself [21]. Although it is unlikely that this approach would be considered in practice for estimating risk differences, we have included it in the current study for comparative purposes.

## 3. Monte Carlo simulations

We used Monte Carlo simulations to examine the performance of different propensity-score methods. We examined Type I error, bias, variance estimation, coverage of confidence intervals, and mean-squared error (MSE).

### 3.1. Methods

We randomly generated data so that they would be similar to the data considered in the case study in Section 4. In particular, we simulated data so that approximately 25 per cent of the sample was exposed to the treatment. Furthermore, we simulated data such that the probability of the outcome would be approximately 0.29 if all subjects in the population were not exposed. We then examined scenarios in which the risk differences between the treated and the untreated subjects were 0, −0.02, −0.05, −0.10, and −0.15 (i.e. absolute reductions in the probability of the outcome due to treatment were 0, 0.02, 0.05, 0.10, and 0.15).

As alluded to in Section 2.4, it is difficult to use a conditional data-generating process to generate binary outcomes and exposure such that the treatment causes a specific risk difference. Our data-generating process used the fact that risk differences are collapsible: the average subject-specific risk difference is equal to the population or marginal risk difference [22]. We used a recently described data-generating process for simulating data in which treatment induces a specified risk difference [23]. This is a modification of a data-generating process for inducing marginal odds ratios of specific magnitudes that has been described elsewhere [7, 24]. We describe this method briefly.

First, we randomly generated 10 independent covariates (*X*_1_ − *X*_10_) from a standard normal distribution for each of 10 000 subjects. We then assumed that the following logistic regression model related the probability of the outcome to these covariates and an indicator variable (*Z*) denoting treatment:


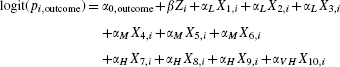
(4)

In the above regression model, *p_i,outcome_* denotes the probability of the outcome for the *i*th subject and β denotes the log-odds ratio relating treatment to the outcome. The regression coefficients for the baseline covariates were set as follows: α_*L*_ = log(1.1), α_*M*_ = log(1.25), α_*H*_ = log(1.5), and α_*V H*_ = log(2). These are intended to reflect low, medium, high, and very high sizes. We fixed the value of α_0,outcome_ = log(0.29/0.71) so that the probability of the event occurring in the population if all subjects were untreated would be approximately 0.29 (to reflect the scenario observed in the case study in Section 4). For a fixed value of β, we determined the model-based predicted probability of the outcome for each subject assuming that the entire population was untreated. The average predicted probability of the outcome if all subjects are untreated is referred to as the marginal probability of the outcome if untreated. We then determined the model-based predicted probability of the outcome for each subject assuming that the entire population was treated. The average predicted probability of the outcome if all subjects are treated is referred to as the marginal probability of the outcome if treated. The difference between the two marginal probabilities is the marginal (or population-average) risk difference. Since the risk difference is collapsible, the marginal risk difference is equal to the average subject-specific risk difference [22]. We repeated the above process 1000 times and determined the average risk difference over 1000 simulated data sets. We then used an iterative process described elsewhere [23], to select the value of β that resulted in the desired non-null risk difference. For a risk difference of 0, β was set to 0. For risk differences of −0.02, −0.05, −0.10, and −0.15, the required value of β equaled 0.8935633, 0.7493734, 0.5446253, and 0.3769236, respectively. Note that since we are estimating marginal or population-average risk differences, the value of β selected will depend on the distribution of baseline covariates in the population.

For each risk difference (0, −0.02, −0.05, −0.10, and −0.15), we randomly generated 1000 data sets with the required risk difference. The different propensity-score methods described in Section 2 were used to estimate the risk difference due to treatment. For propensity-score matching, we matched subjects on the logit of the propensity score using a caliper of width equal to 0.2 of the standard deviation of the logit of the propensity score [25, 26]. We used three different versions of the doubly robust estimator IPTW-DR. The first used the correctly specified regression model to predict outcomes (regressed outcomes on treatment and *X*_1_ − *X*_10_). We refer to this method as IPTW-DR-1. The second version used a mis-specified version of the outcomes regression model. In this model, the outcome was regressed on an indicator for treatment status and the six variables that had a low or moderate effect on the outcome (*X*_1_ − *X*_6_). The four variables that had a high or very high effect on the outcome were omitted from this regression model. We refer to this method as IPTW-DR-2. The third version also used a mis-specified version of the outcomes regression model. In this model, the outcome was regressed only on an indicator variable denoting treatment status. We refer to this method as IPTW-DR-3. We also estimated the crude (unadjusted) risk difference in each simulated data set.

For each true risk difference, we calculated the mean estimated risk difference across the 1000 simulated data sets. We also calculated the mean estimated standard error of the estimated risk differences across the 1000 data sets and the proportion of estimated 95 per cent confidence intervals that contained the true risk difference. We computed the MSE of the estimate. We also determined the ratio of the mean estimated standard error across the 1000 simulated data sets to the standard deviation of the estimated risk differences across the 1000 simulated data sets. When the true risk difference was 0 (null treatment effect), we estimated the empirical type I error rate as the proportion of simulated data sets in which the null hypothesis that the risk difference was equal to zero was rejected at a 0.05 significance level.

### 3.2. Results

The mean estimated risk difference, mean estimated standard error, empirical coverage rates of 95 per cent confidence intervals, MSE, and ratio of mean standard error of estimates to the standard deviation of the estimated risk differences are reported in Table I for each of the different propensity-score methods and for each of the five different true risk differences. We discuss the performance of the different propensity-score methods on each of these metrics in the following paragraphs.

**Table I:** Results of Monte Carlo simulations examining performance of different propensity-score methods for estimating risk differences

True risk difference	Crude risk difference	Covariate adjustment	Stratification	Matching	IPTW1	IPTW-DR-1	IPTW-DR-2	IPTW-DR-3
*Mean estimated risk difference across 1000 simulated data sets*								
0	0.181	0.000	0.015	0.001	0.000	0.000	0.000	0.000
−0.02	0.158	−0.023	−0.005	−0.023	−0.020	−0.020	−0.020	−0.020
−0.05	0.122	−0.057	−0.035	−0.058	−0.050	−0.050	−0.050	−0.050
−0.1	0.060	−0.115	−0.086	−0.119	−0.100	−0.100	−0.100	−0.100
−0.15	−0.005	−0.174	−0.137	−0.182	−0.150	−0.150	−0.150	−0.150
*Mean estimated standard error across 1000 simulated data sets*								
0		0.010	0.011	0.013	0.013	0.010	0.013	0.013
−0.02		0.010	0.010	0.013	0.013	0.010	0.012	0.013
−0.05		0.010	0.010	0.013	0.012	0.010	0.012	0.013
−0.1		0.010	0.010	0.013	0.012	0.009	0.011	0.012
−0.15		0.010	0.009	0.012	0.011	0.009	0.010	0.011
*Empirical coverage rates of 95 per cent confidence intervals across 1000 simulated data sets*								
0		0.933	0.699	0.936	0.978	0.953	0.978	0.982
−0.02		0.938	0.710	0.948	0.979	0.959	0.976	0.984
−0.05		0.890	0.685	0.902	0.987	0.962	0.982	0.984
−0.1		0.678	0.691	0.665	0.979	0.955	0.971	0.977
−0.15		0.309	0.675	0.228	0.991	0.964	0.985	0.986
*Mean-squared error (MSE) of estimated risk differences*								
0	0.032893	0.000119	0.000337	0.000168	0.000112	0.000109	0.000119	0.000121
−0.02	0.031836	0.000120	0.000327	0.000166	0.000103	0.000100	0.000109	0.000112
−0.05	0.029782	0.000160	0.000309	0.000227	0.000096	0.000094	0.000102	0.000104
−0.1	0.025811	0.000335	0.000284	0.000531	0.000087	0.000084	0.000087	0.000088
−0.15	0.021057	0.000689	0.000252	0.001186	0.000076	0.000071	0.000074	0.000074
*Ratio of mean standard error of estimate to standard deviation of estimated risk differences*								
0		0.943	0.996	0.999	1.248	1.000	1.170	1.221
−0.02		0.973	1.019	1.019	1.270	1.020	1.189	1.238
−0.05		0.988	1.024	1.018	1.267	1.018	1.184	1.233
−0.1		1.007	1.016	0.995	1.242	1.015	1.184	1.237
−0.15		1.053	1.029	1.021	1.235	1.028	1.177	1.223

One observes that the four estimators that used IPTW resulted in essentially unbiased estimation of the true risk difference. Matching on the propensity score resulted in estimated risk differences that were modestly biased away from the null treatment effect: the estimates displayed a greater absolute risk reduction compared with the true absolute risk reduction. Similarly, stratification on the quintiles of the propensity score resulted in estimated risk differences that displayed modest bias. However, for stratification, the bias was toward the null treatment effect. Finally, covariate adjustment using the propensity score resulted in estimates that displayed a bias similar to that of propensity-score matching. When the true risk difference was different from 0, propensity-score matching resulted in relative biases ranging from 15 to 21 per cent, with the relative bias increasing with the true risk difference. Stratification on the quintiles of the propensity score resulted in relative biases ranging from −9 to −75 per cent, with relative bias increasing in absolute value as the true risk difference decreased in absolute magnitude. Covariate adjustment using the propensity score resulted in relative biases ranging from 14 to 16 per cent.

For a given true risk difference, the mean standard error of the estimated risk difference was minimized when propensity-score weighting using the doubly robust estimator and the correctly specified outcomes model (IPTW-DR-1) was used. For some values of the true risk difference, either covariate adjustment using the propensity score or stratification on the quintiles of the propensity score resulted in estimates with the same mean standard error as IPTW-DR-1. For a given true risk difference, the mean standard error of the estimated risk difference was largest when propensity-score matching was employed. For some values of the true risk difference, IPTW1, IPTW-DR-2, or IPTW-DR-3 had estimates with the mean standard error as large as that of propensity-score matching.

IPTW-DR-1 had 95 per cent confidence intervals whose coverage rates ranged from 0.953 to 0.964 across the five different scenarios. The other methods based on IPTW resulted in confidence intervals that were conservative—the coverage rates of the 95 per cent confidence intervals exceeded the advertised rate. For propensity-score matching, the empirical coverage rates were approximately correct when the true risk difference was 0 or −0.02. However, it was substantially lower for larger risk differences. Stratification on the quintiles of the propensity score always produced 95 per cent confidence intervals whose empirical coverage rates were at most 0.710. Covariate adjustment using the propensity score resulted in confidence intervals with coverage rates similar to those of propensity-score matching.

For all values of the true risk difference, IPTW-DR-1 resulted in estimates with the lowest MSE. However, in all instances, the other estimates based on IPTW had very similar estimates to that of the IPTW-DR-1 estimate. Stratification on the quintiles of the propensity score resulted in estimates with MSE that were approximately 3 to 3.5 times greater than those of IPTW-DR-1. Matching resulted in estimates whose MSE was between 1.5 and 17 times greater than those of IPTW-DR-1.

For propensity-score matching, the ratio between the mean standard error of the estimated risk differences and the standard deviation of the estimated risk differences across the 1000 simulated data sets ranged from 0.995 to 1.021, indicating that the sampling variability of the estimated risk difference was well approximated by the estimated standard error of the risk difference. Similarly, for the IPTW-DR-1 estimator, this ratio ranged from 1.000 to 1.028. However, for IPTW1, IPTW-DR-2, and IPTW-DR-3, this ratio ranged from 1.170 to 1.270, indicating that the estimated standard error of the estimated risk difference was over-estimating the standard deviation of the sampling distribution by approximately 17 to 27 per cent. For stratification, this ratio ranged from 0.996 to 1.029, whereas for covariate adjustment using the propensity score the ratio ranged from 0.943 to 1.05. Thus, for stratification, the estimated standard error of the estimated risk difference tended to closely approximate the standard deviation of the sampling distribution of the estimated risk difference.

When the true risk difference was equal to 0, the empirical type I error rates were estimated by the proportion of simulated data sets in which the null hypothesis of a null risk difference was rejected. The empirical type I error rates were as follows: covariate adjustment using the propensity score: 0.067; stratification on the quintiles of the propensity score: 0.301; propensity-score matching: 0.064; IPTW1: 0.022; IPTW-DR-1: 0.047; IPTW-DR-2: 0.022; and IPTW-DR-3: 0.018. Owing to our use of 1000 simulated data sets, any empirical type I rate that is higher than 0.0635 or lower than 0.0365 would be significantly different from 0.05 using a standard normal approximation to the binomial distribution test. Thus, only the IPTW-DR-1 estimator had an observed empirical type I error rate that was not significantly different from 0.05. All the other propensity-score methods had empirical type I error rates that were significantly different from 0.05. However, propensity-score matching and covariate adjustment using the propensity score had empirical type I error rates that were approximately equal to the nominal level. The other IPTW estimators had empirical type I error rates that were less than 0.05 (0.018 to 0.022).

## 4. Case study

### 4.1. Data sources

Detailed clinical data were obtained by a retrospective chart review on a sample of 7613 patients discharged alive with a diagnosis of heart failure between 1 April 1999 and 31 March 2001 from 103 acute care hospitals in Ontario, Canada. Further detail of the data obtained is provided elsewhere [27, 28]. These data were collected as a part of the Enhanced Feedback for Effective Cardiac Treatment (EFFECT) Study, an ongoing initiative intended to improve the quality of care for patients with cardiovascular disease in Ontario [28]. Data on patient demographics, vital signs at presentation, and results of physical examination at presentation, medical history, and results of laboratory tests were collected for this sample. Subjects with missing data on key continuous baseline covariates were excluded from the current study. In the current study, we examined receipt of a prescription for a beta-blocker at discharge as the exposure of interest. The demographic and the clinical characteristics of the treated and the untreated subjects are described in Table II. Continuous and categorical variables were compared between the treated and the untreated subjects using the Wilcoxon Rank Sum test and the Chi-squared test, respectively. Standardized differences are also reported for comparing the mean of variables between treatment groups [29]. Systematic differences in several variables, including age, systolic blood pressure, heart rate, history of previous myocardial infarction, history of chronic obstructive pulmonary disease (COPD), and dementia, were observed between the treatment groups. Overall, 27.3 per cent of patients received a prescription for a beta-blocker at discharge. The outcome of interest was death within 1 year of hospital discharge. 27.7 per cent of the subjects died within 1 year of hospital discharge.

**Table II:** Baseline characteristics of beta-blocker and non-beta-blocker patients in the case study

Baseline characteristics	Beta-blocker: no (*N* = 5535)	Beta-blocker: yes (*N* = 2078)	Standardized difference	*P*-value
	Median (25th percentile - 75th percentile) or *N* (per cent)			
*Demographic characteristics*				
Age, years	78 (70–84)	75 (67–82)	0.24	<0.001
Female	2809 (50.7 per cent)	1011 (48.7 per cent)	0.04	0.103
*Vital signs on admission*				
Systolic blood pressure, mmHg	147 (127–170)	150 (130–176)	0.13	<0.001
Heart rate, beats per minute	94 (78–111)	88 (73–108)	0.14	<0.001
Respiratory rate, breaths per minute	24 (20–30)	24 (20–28)	0.09	<0.001
*Presenting symptoms and physical exam*				
Neck vein distension	3002 (54.2 per cent)	1200 (57.7 per cent)	0.07	0.006
S3	518 (9.4 per cent)	232 (11.2 per cent)	0.06	0.018
S4	204 (3.7 per cent)	89 (4.3 per cent)	0.03	0.227
Rales > 50 per cent of lung field	560 (11.1 per cent)	231 (11.1 per cent)	0.03	0.203
*Findings on chest X-Ray*				
Pulmonary edema	2772 (50.1 per cent)	1137 (54.7 per cent)	0.09	<0.001
Cardiomegaly	2026 (36.6 per cent)	711 (34.2 per cent)	0.05	0.053
*Past medical history*				
Diabetes	1871 (33.8 per cent)	804 (38.7 per cent)	0.1	<0.001
CVA/TIA	880 (15.9 per cent)	340 (16.4 per cent)	0.01	0.624
Previous MI	1815 (32.8 per cent)	989 (47.6 per cent)	0.31	<0.001
Atrial fibrillation	1675 (30.3 per cent)	530 (25.5 per cent)	0.1	<0.001
Peripheral vascular disease	684 (12.4 per cent)	302 (14.5 per cent)	0.06	0.012
Chronic obstructive pulmonary disease	1074 (19.4 per cent)	191 (9.2 per cent)	0.28	<0.001
Dementia	422 (7.6 per cent)	91 (4.4 per cent)	0.13	<0.001
Cirrhosis	48 (0.3 per cent)	6 (0.3 per cent)	0.07	0.007
Cancer	659 (11.9 per cent)	195 (9.4 per cent)	0.08	0.002
*Electrocardiogram—first available within 48 h*				
Left bundle branch block	834 (15.1 per cent)	293 (14.1 per cent)	0.03	0.29
*Laboratory tests*				
Hemoglobin, g/L	124 (110–138)	125 (111–139)	0.05	0.146
White blood count, 10E9/L	9 (7–12)	9 (7–11)	0.02	0.261
Sodium, mmol/L	139 (136–141)	139 (137–141)	0.08	0.001
Potassium, mmol/L	4 (4–5)	4 (4–5)	0.03	0.12
Glucose, mmol/L	7(6–11)	8 (6–12)	0.09	<0.001
Blood urea nitrogen, mmol/L	8 (6–12)	8 (6–12)	0	0.522
Creatinine, μmol/L	104 (82–142)	107 (85–144)	0.08	0.002

### 4.2. Statistical analyses

An indicator variable denoting receipt of a beta-blocker prescription at hospital discharge was regressed on the 28 baseline characteristics described in Table II using a logistic regression model. The estimated propensity score was the predicted probability of receiving a beta-blocker prescription that was derived from the fitted logistic regression model.

Once the estimated propensity score had been obtained, the different propensity-score methods described in Section 2 were used to estimate the absolute reduction in the probability of death within one year of discharge due to receipt of a beta-blocker prescription. The associated NNT was the reciprocal of the absolute risk reduction. For propensity-score matching, the treated and the untreated subjects were matched on the logit of the propensity score using calipers of width equal to 0.2 of the standard deviation of the logit of the propensity score. We used two different doubly robust estimators using IPTW. The first used all 28 variables listed in Table II to predict mortality. The second used a regression model that only contained an indicator variable for treatment with beta-blocker at hospital discharge.

### 4.3. Results

The estimated reduction in the probability of 1-year mortality for each of the propensity-score methods is reported in Table III. The absolute reduction in the probability of death within one year of discharge due to receipt of a beta-blocker prescription ranged from a low of 0.047 (4.7 per cent) for propensity-score matching to a high of 0.053 (5.3 per cent) for stratification on the quintiles of the propensity score. The estimated absolute risk reductions were qualitatively similar across the different propensity-score methods. The associated numbers needed to treat ranged from a low of 18.9 (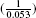) for stratification on the propensity score to a high of 21.3 (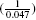) for propensity-score matching. The three different methods that used IPTW produced very similar estimates of the absolute risk reduction (0.050 to 0.051).

**Table III:** Estimated absolute risk reduction in case study

Propensity score method	Absolute risk reduction	Ninety-five per cent confidence interval for the absolute risk reduction
Covariate adjustment using the propensity score	0.05	(0.027, 0.073)
Stratification on the propensity score	0.053	(0.03, 0.077)
IPTW1	0.051	(0.024, 0.078)
IPTW-DR-1	0.05	(0.028, 0.073)
IPTW-DR-2	0.051	(0.026, 0.075)
Propensity-score matching	0.047	(0.022, 0.073)

The doubly robust IPTW estimator with the full outcome-regression model had the narrowest 95 per cent confidence interval (width = 0.045). The simple IPTW1 estimator had a 95 per cent confidence interval that was 20 per cent wider. Propensity-score matching resulted in a 95 per cent confidence interval that was 13 per cent wider than that of the doubly robust method with the fully specified outcomes regression model. Similarly, stratification on the quintiles of the propensity score resulted in a 95 per cent confidence interval that was 4 per cent wider than that of the doubly robust methods.

## 5. Discussion

In this paper we used Monte Carlo simulations to examine the performance of different propensity-score methods for estimating risk differences. The estimators based on using IPTW resulted in unbiased estimation of the risk differences. The other propensity-score methods introduced minor to modest bias. The IPTW doubly robust estimator with the correctly specified outcomes regression model resulted in estimates with the lowest estimated standard errors. Similarly, this method resulted in estimates with the lowest MSE, although the other IPTW estimators were close competitors. The IPTW doubly robust estimator with the correctly specified outcomes regression model resulted in confidence intervals with the advertised coverage rates, whereas those of other IPTW estimators had coverage rates that exceeded the advertised levels. In some scenarios, propensity-score matching resulted in confidence intervals whose coverage rates were substantially lower than the advertised levels. Similarly, the IPTW doubly robust estimator with the correctly specified outcomes regression model resulted in approximately correct type I error rates, whereas the other IPTW estimators had type I error rates lower than the nominal level. Finally, we observed that the standard errors for stratification, matching, and the doubly robust IPTW estimator with the correctly specified regression model closely approximated the standard deviation of the sampling distribution of the estimators.

In our case study, we observed that the different propensity-score methods resulted in qualitatively similar estimates of the absolute reduction in the probability of mortality within 1 year due to receipt of a beta-blocker prescription at hospital-discharge. Similarly, estimates of the NNT to avoid one death within 1 year were qualitatively similar across the different methods (range 19–21). IPTW using the doubly robust estimator with the full regression model resulted in a 95 per cent confidence interval with the narrowest width. Propensity-score matching resulted in a 95 per cent confidence interval that was 13 per cent wider than the doubly robust method.

When outcomes are binary, measures of treatment effect can be reported using odds ratios, relative risks, or risk differences. Several studies have examined the performance of different propensity-score methods for estimating relative risks and odds ratios. Austin *et al.* found that propensity-score methods result in biased estimation of conditional or adjusted odds ratios [6]. Furthermore, propensity-score matching, stratification on the propensity score, and covariate adjustment using the propensity score result in sub-optimal inferences about marginal or population-average odds ratios [7]. However, propensity-score methods allow for unbiased estimation of relative risks in the presence of a uniform treatment effect [8]. When used for estimating relative risks, stratification and matching displayed the variance–bias trade-off: matching resulted in estimates with less bias, whereas stratification resulted in estimates with lower variance and greater precision [8]. In contrast to these prior studies examining estimation of odds ratios and relative risks, there is a paucity of information on the performance of different propensity-score methods for estimating risk differences.

Clinical commentators have suggested that absolute risk reductions and numbers needed to treat provide important information for clinical decision making that is lacking in relative measures of effect such as the odds ratio and the relative risk [1–4].

Furthermore, some medical journals require that the NNT be reported for any randomized clinical trial with binary outcomes [5]. These clinically meaningful measures of treatment effect can be easily computed using propensity-score methods. The current study provides the first comprehensive examination of the performance of the four different propensity-score methods for estimating risk differences. Given the advantages of the absolute risk reduction and the NNT for clinical decision making, we suggest that these measures of effect should also be reported for any observational study with binary outcomes.

Based on the results of our Monte Carlo simulations, IPTW using the doubly robust estimator had the superior performance of the different propensity-score methods examined. It resulted in essentially unbiased estimation of the true risk difference, had the lowest standard error of the estimated risk difference, had the lowest MSE, resulted in 95 per cent confidence intervals with approximately correct coverage rates, and had approximately correct type I error rates. Each of the competing approaches had inferior performance on at least one of these metrics compared with the doubly robust approach. A limitation of the doubly robust approach is the requirement that one specify an outcomes regression model relating the outcome to baseline covariates. However, we found that if the outcomes model was mis-specified through the omission of several predictor variables, then superior performance was still achieved relative to stratification or matching on the propensity score. Propensity-score matching and stratification on the quintiles of the propensity score are two commonly used approaches in the medical literature, whereas IPTW methods are rarely employed in the medical literature [19, 20]. When the comparison was restricted to stratification and matching, one observed that matching had superior performance for low to moderate effect sizes, whereas stratification had superior performance for larger effect sizes. However, matching resulted in an approximately correct type I error rate, whereas stratification had a substantially inflated type I error rate.

There has been limited comparison of methods that employ IPTW with other propensity-score methods in the literature. Lunceford and Davidian [18] compared methods employing weighting via the propensity score with stratification in the context of a continuous outcome and a linear treatment effect. Some of our observations mirror their findings. For instance, they note that stratification is not a consistent estimator, resulting in biased estimation of linear treatment effects. Furthermore, they note that the IPTW-DR-1 estimator has lower variance than the IPTW1 estimator, which reflects the findings of our Monte Carlo simulations. In addition, Lunceford and Davidian observed that stratification resulted in estimates with higher MSE compared with IPTW-DR-1, and that confidence intervals for stratified estimators did not have the advertised coverage rates. The current study made two novel contributions. The first was the focus on estimating risk differences rather than differences in means. In randomized controlled trials of medical interventions, binary outcomes are more prevalent than continuous outcomes [30]. Therefore, our focus on estimating risk differences may provide more guidance to medical researchers examining the effects of treatments on health outcomes using observational data. The second novel contribution was the inclusion of propensity-score matching and covariate adjustment using the propensity score. Both of these methods are used more frequently than methods based on IPTW [19, 20]. Propensity-score matching is frequently used in the medical literature. It is important to determine its relative performance compared with the competing methods for estimating risk differences.

The propensity score is a balancing score: conditional on the propensity score, the distribution of measured baseline covariates is similar between the treated and the untreated subjects [9]. Several recent studies have compared the relative ability of different propensity-score methods with balance measured baseline covariates between the treated and the untreated subjects. Propensity-score matching has been shown to eliminate a greater proportion of the systematic differences between the treated and the untreated subjects compared with stratification on the propensity score [25, 26, 31]. Similarly, propensity-score matching eliminated a greater degree of the systematic differences between the treated and the untreated subjects compared with covariate adjustment using the propensity-score [31]. Finally, in some settings, propensity-score matching and IPTW using the propensity score eliminated systematic differences between the treated and the untreated subjects to an approximately equivalent degree [31]. However, there were some scenarios in which propensity-score matching eliminated a modestly greater proportion of the observed imbalance compared with IPTW using the propensity score [31].

A limitation to the use of methods based on IPTW is the paucity of methods that have been described in the literature for assessing whether the propensity-score model has been correctly specified in this context. When stratification or matching on the propensity score is employed, a range of diagnostics have been described for assessing the adequacy of the specification of the propensity-score model [10, 25, 26, 32]. However, there are limited descriptions of methods to assess the goodness-of-fit of the propensity-score model in the context of IPTW (one assumes that many methods for matching could be adapted to the use of IPTW using the propensity score). Rubin writes ‘*In rare situations, the individually estimated probabilities (i.e. the estimated propensity scores) themselves may be used in the process of estimating treatment effects … If it is, the propensity-score estimation has to be conducted far more carefully. … In such cases, the estimated probabilities can be very influential on the estimated effects of treatment versus control, and so the probabilities themselves must be very well-estimated. In such cases, diagnostics of the accuracy of the estimated probabilities are appropriate, although diagnostics of the estimated underlying (logistic) regression coefficients are generally irrelevant*’ [33]. Thus, a prelude to the greater use of methods based on IPTW using the propensity score may be the development of diagnostics for assessing the accuracy of the estimated propensity scores.

The apparent superiority of IPTW using the propensity score compared with propensity-score matching may be worrisome, given the popularity of the latter method [12–14]. However, a possible explanation for the discrepancies between these two methods is that they are estimating different measures of effect. The econometrics literature differentiates between the average treatment effect (ATE) and the average treatment effect for the treated (ATT) [34]. Imbens [34] states that stratification using the propensity score and IPTW using the propensity score allow one to estimate the ATE, whereas matching on the propensity score allows one to estimate the ATT. The data-generating process in the current study induced a specified ATE. Thus, the bias estimation that arose when using propensity-score matching may primarily be a result of the fact that matching estimates the ATT, whereas stratification and weighting estimate the ATE.

In conclusion, our study suggests that a greater use of methods based on IPTW should be used for estimating risk differences in observational studies. This is particularly true when the interest is in estimating ATEs. Although the focus in the past has been on odds ratios and relative risks, estimation of absolute risk reductions and numbers needed to treat may provide greater information for clinical decision making.
